# Lower Interferon Regulatory Factor-8 Expression in Peripheral Myeloid Cells Tracks With Adverse Central Nervous System Outcomes in Treated HIV Infection

**DOI:** 10.3389/fimmu.2019.02789

**Published:** 2019-11-29

**Authors:** Michelle L. D'Antoni, Kalpana J. Kallianpur, Thomas A. Premeaux, Michael J. Corley, Tsuyoshi Fujita, Elizabeth I. Laws, Debra Ogata-Arakaki, Dominic C. Chow, Vedbar S. Khadka, Cecilia M. Shikuma, Lishomwa C. Ndhlovu

**Affiliations:** ^1^Department of Tropical Medicine, University of Hawai'i, Honolulu, HI, United States; ^2^Hawaii Center for AIDS, University of Hawai'i, Honolulu, HI, United States; ^3^Department of Native Hawaiian Health, University of Hawai'i, Honolulu, HI, United States; ^4^Office of Biostatistics and Quantitative Health Sciences, John A. Burns School of Medicine, University of Hawai'i, Honolulu, HI, United States

**Keywords:** HIV-1, IRF-8, interferon, monocytes, dendritic cells, cognition, central nervous system, anti-retroviral therapy

## Abstract

Cognitive dysfunction persists in 30–50% of chronically HIV-infected individuals despite combination antiretroviral therapy (ART). Although monocytes are implicated in poor cognitive performance, distinct biological mechanisms associated with cognitive dysfunction in HIV infection are unclear. We previously showed that a regulatory region of the *interferon regulatory factor-8* (*IRF8*) gene is hyper-methylated in HIV-infected individuals with cognitive impairment compared to those with normal cognition. Here, we investigated IRF-8 protein expression and assessed relationships with multiple parameters associated with brain health. Intracellular IRF-8 expression was measured in cryopreserved peripheral blood mononuclear cells from chronically HIV-infected individuals on ART using flow cytometry. Neuropsychological performance was assessed by generating domain-specific standardized (NPZ) scores, with a global score defined by aggregating individual domain scores. Regional brain volumes were obtained by magnetic resonance imaging and soluble inflammatory factors were assessed by immunosorbent assays. Non-parametric analyses were conducted and statistical significance was defined as *p* < 0.05. Twenty aviremic (HIV RNA<50 copies/ml) participants, 84% male, median age 51 [interquartile range (IQR) 46, 55], median CD4 count 548 [439, 700] were evaluated. IRF-8 expression was highest in plasmacytoid dendritic cells (pDCs). Assessing cognitive function, lower IRF-8 density in classical monocytes significantly correlated with worse NPZ_learning memory (LM; rho = 0.556) and NPZ_working memory (WM; rho = 0.612) scores, in intermediate monocytes with worse NPZ_LM (rho = 0.532) scores, and in non-classical monocytes, lower IRF-8 correlated with worse global NPZ (rho = 0.646), NPZ_LM (rho = 0.536), NPZ_WM (rho = 0.647), and NPZ_executive function (rho = 0.605) scores. In myeloid DCs (mDCs) lower IRF-8 correlated with worse NPZ_WM (rho = 0.48) scores and in pDCs with worse NPZ_WM (rho = 0.561) scores. Declines in IRF-8 in classical monocytes significantly correlated with smaller hippocampal volume (rho = 0.573) and in intermediate and non-classical monocytes with smaller cerebral white matter volume (rho = 0.509 and rho = 0.473, respectively). IRF-8 density in DCs did not significantly correlate with brain volumes. Among biomarkers tested, higher soluble ICAM-1 levels significantly correlated with higher IRF-8 in all monocyte and DC subsets. These data may implicate IRF-8 as a novel transcription factor in the neuropathophysiology of brain abnormalities in treated HIV and serve as a potential therapeutic target to decrease the burden of cognitive dysfunction in this population.

## Introduction

Cognitive performance is compromised in ~30–50% of chronically HIV-infected individuals despite access to combination antiretroviral therapy (ART) (1, 2). These cognitive, behavioral, and motor deficits are not only widespread, but also impact everyday functioning, increase morbidity and mortality and have lasting critical public health effects (3–5). Since clinically approved therapies for HIV-associated cognitive impairment are not available (6), there is need to identify novel therapeutic targets.

The transmigration of both infected and uninfected monocytes into the central nervous system (CNS) is thought to be a significant mediator of the development of cognitive disorders during HIV infection, primarily by promoting viral seeding of CNS resident cells and promoting neuroinflammatory responses (7–14). Furthermore, myeloid cells, including monocytes and dendritic cells (DCs), play crucial roles in maintaining homeostasis along with inducing and controlling neuroinflammatory responses when recruited to the CNS (15–17). The implications of myeloid cells entering the CNS can differ depending on the pathological state and their peripheral phenotype, either inducing neuroinflammatory cytotoxic effects or promoting neural regeneration (18–20). Therefore, a better understanding of molecular mechanisms linking peripheral myeloid cells to the CNS is crucial to further elucidate the pathophysiology of HIV-associated brain injury.

Previously, we identified 1,032 differentially methylated loci in monocytes from persons with and without HIV-associated cognitive impairment. *IRF8*, the gene encoding for interferon regulatory factor-8 (IRF-8), had regions of significant hypermethylation in HIV-infected individuals with cognitive impairment compared to those with normal cognition, suggesting a potential role for this otherwise constitutively expressed transcription factor in HIV-related cognitive dysfunction (21). IRF-8 plays critical roles in the regulation of lineage commitment and differentiation during myeloid cell maturation and response to stimuli (22–24). For example, IRF-8 expression is elevated in the brains of a Alzheimer's Disease rodent model and in the context of accelerated aging and Alzheimer's Disease, IRF-8 was found to be one of the biomarkers with the highest correlation coefficient (25–27). Here, we wish to extend our *IRF8* epigenetic findings and evaluate IRF-8 protein expression in myeloid cells and the relationship to several measures of cognition, CNS injury, and inflammation in virally suppressed chronic HIV individuals on stable ART.

## Materials and Methods

### Cohort Description

This study consisted of 20 chronically HIV infected individuals from the Hawaii Aging with HIV—Cardiovascular cohort study. The study, which has been previously described (28), was approved by the University of Hawaii Manoa Committee on Human Studies. Entry criteria to the study required subjects to have documentation of HIV infection, be ≥40 years, and to be on stable ART for≥6 months. Two study participants had a history of Hepatitis C infection. The selection of participants for our study was based on the availability of neuropsychological (NP) testing data and banked cryopreserved peripheral blood mononuclear cells (PBMCs) and plasma.

### Neuropsychological Assessments

NP testing was conducted at the UH Clint Spencer Clinic by trained psychometrists. In order to minimize the risk of distraction and fatigue, NP testing was conducted in a quiet room and participants were provided breaks as needed throughout the testing session. The test battery was comprised of measures known to be sensitive to HIV infection including psychomotor speed, executive function, learning and memory, and working memory (29, 30). All raw NP scores were transformed to standard z-scores (NPZ score) using normative data (31, 32) and a global score was defined by aggregating the domain scores.

### Regional Brain Volume Assessments by Magnetic Resonance Imaging

T1-weighted MRI data were processed with FreeSurfer (version 5.0, https://surfer.nmr.mgh.harvard.edu) (33–36) to obtain volumes of the caudate, putamen, hippocampus, amygdala, cortical gray matter, cerebral white matter, cerebellar gray matter, cerebellar white matter, and total subcortical gray matter, FreeSurfer's processing steam involves skull-stripping (37), intensity normalization (38), Talairach transformation, segmentation of subcortical white matter and deep gray matter (34, 35), and reconstruction of the cortical gray/white matter boundary and pial surface (33). Total regional volumes were computed by summing over the left and right hemispheres. An estimate of total intracranial volume (ICV) was obtained and used to normalize the regional volumes of interest (39). Each regional volume was expressed as a fraction of ICV (i.e., volume/ICV) to adjust for inter-individual head size variability.

### IRF-8 Intracellular Staining

Cryopreserved PBMCs were placed in 96 well-polypropylene round bottom plates and stained with Live/Dead® Fixable Red Dead Cell Stain for 15 min at room temperature followed by a 30 min room temperature with conjugated monoclonal antibodies (mAbs) against CD3 [Brilliant Violet (BV)711], CD4 (PE-Texas Red), CD8 (PE-Cy5), CD7 (PE-Cy7), CD19 (PE-Cy7), CD20 (PE-Cy7), CD11c (AlexaFluor700), CD123 (FITC), CD11b (BV510), HLA-DR (APC-H7), CD14 (BV605), CD16 (BV421). Cells were then fixed and permeabilized with BD FACS Lysing Solution and Permeabilizing Solution 2 (BD Bioscience, San Jose, USA), respectively, then stained with an anti-IRF-8 antibody or isotype control (PerCP-eF710). Cells were fixed with 1% PFA and samples were acquired on a custom 4-laser BD LSRFortessa (BD Bioscience, San Jose, USA). Compensation and gating analyses were performed using FlowJo (FlowJo LCC, Ashland, USA). Reagents were purchased from BD Bioscience, San Jose, USA (mAbs CD123 Catalog Number [Cat] 558663, CD8 Cat 555368, CD19 Cat 557835, CD20 Cat 560735), Invitrogen, Carlsbad, USA (Live/Dead Stain, CD4 Cat MHCD0417), BioLegend, San Diego, USA (mAbs CD3 Cat 317328, CD7 Cat 343113, CD11b Cat 301333, HLA-DR Cat 307618, CD14 Cat 301834, CD16 Cat 302038) and eBioscience, San Diego, USA (mAbs CD11c Cat 56-0116-42, IRF-8 Cat 46-9852-80, isotype control Cat 46-4714-82). The implemented phenotyping gating strategy is shown in Supplementary Figure 1.

### Quantification of Plasma Markers

The plasma soluble biomarkers, matrix metallopeptidase-9, myeloperoxidase, tissue plasminogen activator inhibitor-1, C-reactive protein, serum amyloid A, serum amyloid P, interleukin (IL)-1β, IL-6, IL-8, IL-10, tumor necrosis factor (TNF)-α, soluble E-selectin, soluble vascular cell adhesion molecule-1, soluble intercellular cell adhesion molecule-1 (sICAM-1), monocyte chemoattractant protein-1 (MCP-1), vascular endothelial growth factor, interferon-gamma (IFN-γ), and N-terminal pro-brain natriuretic peptide were measured by Milliplex Human Cardiovascular Disease panels (EMD Millipore, Temecula, CA) as outlined in the manufacturer's protocols as previously described (40).

### Statistical Analysis

Demographic and clinical characteristics were presented as a median and interquartile range (IQR) except for gender, which was presented as a percentage. Comparisons between continuous variables were carried out using Kruskal-Wallis tests, and for categorical variables, chi-squared tests. For IRF-8 outcomes, Kruskal Wallis tests were used to compare between groups. Associations between two continuous variables were evaluated by Spearman correlation. Statistical analyses were performed using R v3.2.2 or Prism GraphPad version 7 (GraphPad Software, San Diego, California).

## Results

### Participant Characteristics and IRF-8 Expression in Blood

Clinical and demographic characteristics of the participants are shown in Table 1. The twenty participants included in this study had undetectable viral loads (<48 copies/ml), were 84% male, with a median age of 51 ([IQR 47, 55]), a median CD4 count of 548 [439, 700]), and had a range of cognitive performance [global score of −0.04 (−0.46, 0.55)]. IRF-8 density in each myeloid subset from HCV-infected and HCV-uninfected subjects are shown in Supplementary Figure 2.

**Table 1:** Characteristics of the study participants.

**Clinical and Demographic**	
**Characteristics of Participants (*n* = 20)**	
Gender (% male)	84% (*n* = 17)
Age (years)	51 [47, 55]
Education (years)	14 [14, 16]
CD4%	30 [25, 36]
CD4 Nadir^*^ (cells/mm^3^)	108 [34, 234]
CD4 Count (cells/mm^3^)	548 [439, 700]
COB Count (cells/mm^3^)	795 [611, 1140]
CD4/CD8 ratio	0.769 [0.491, 0.879]
HIV RNA (copies/ml)	48 [48, 48]
Years Infected^*^	15.7 [11.8, 17.3]
Years on ARV^*^	13.7 [10.8, 16.3]
NPZ Global (NPZ14)	−0.04 [−0.46, 0.55]

Median [IQR] presented for continuous variables and N (%) presented for categorical variables.

* Self-reported.

*Relevant demographic and clinical information of the study participants*.

**Figure 1:**
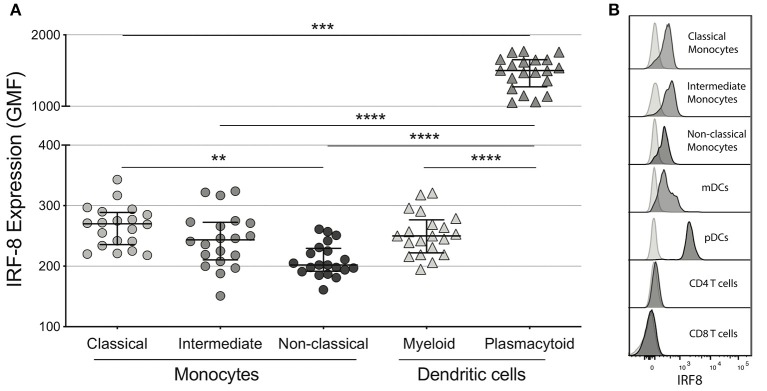
IRF-8 expression on monocytes and dendritic cells. **(A)** Intracellular IRF-8 levels, expressed as geometric mean fluorescence intensity (GMF), on monocyte subsets (classical, intermediate, and non-classical) and dendritic cell (DC) subsets (myeloid and plasmacytoid). The hash mark indicates the median and error bars are the interquartile range. **p < 0.01; ***p < 0.001, ****p < 0.0001. **(B)** Representative histograms of IRF-8 expression in each subset.

Among the myeloid subsets analyzed, plasmacytoid DCs (pDCs) had the highest IRF-8 expression (geometric mean fluorescence intensity (GMF) 1501 [1273, 1651]), which was significantly higher than all 3 monocyte subsets and myeloid DCs (mDCs) (GMF 250 [222,277]) in concordance with a previous murine study (*p*'s <0.001; Figures 1A,B) (41). Non-classical monocytes had the lowest IRF-8 expression (GMF 202 [192, 230]) compared to intermediate monocytes (GMF 244 [211, 273], not significant) and classical monocytes (GMF 270 [236, 289]; *p* < 0.01) (Figures 1A,B), while CD4+ and CD8+ T cells had undetectable IRF-8 levels (Figure 1B). Frequencies of each myeloid subset are show in Supplementary Table 1. IRF-8 expression in all myeloid subsets did not associate with age, CD4 count or nadir, CD8 count, CD4/CD8 ratio, or total self-reported years of infection or on ART (data not shown).

### IRF-8 Density in Myeloid Cells Correlates With Cognitive Performance

**Figure 2:**
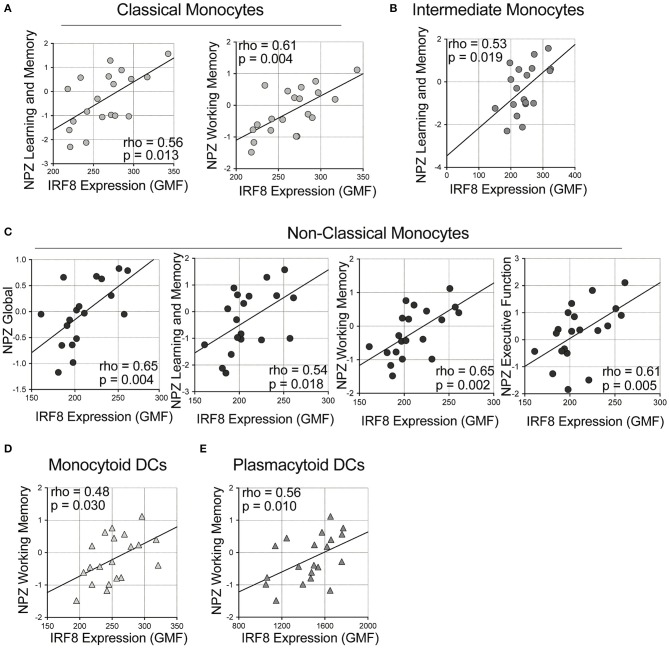
Correlations between IRF-8 expression on myeloid cells and neuropsychological testing. Correlations between IRF-8 expression of **(A)** classical, **(B)** intermediate, and **(C)** non-classical monocytes, and **(D)** myeloid and **(E)** plasmacytoid dendritic cells and NPZ scores.

Next, correlations of IRF-8 density in myeloid cells and neurocognitive functions were analyzed (Figure 2 and Supplementary Table 2). Higher IRF-8 expression on classical monocytes correlated with better NPZ_learning and memory (LM) (rho = 0.556, *p* = 0.013) and working memory (WM) (rho = 0.612, *p* = 0.004; Figure 2A) scores. Higher IRF-8 expression on intermediate monocytes also correlated with better NPZ_LM scores (rho = 0.532, *p* = 0.019; Figure 2B). Greater IRF-8 expression on non-classical monocytes also correlated with increased NP testing (global: rho = 0.646; *p* = 0.004; LM: rho = 0.536, *p* = 0.018, WM: rho = 0.647, *p* = 0.002 and executive function: rho = 0.605; *p* = 0.005; Figure 2C). Higher IRF-8 expression on both mDCs and pDCs also correlated with better NPZ_WM scores (rho = 0.484, *p* = 0.030 and rho = 0.561, *p* = 0.010, respectively; Figures 2D,E). Age did not show significant correlations with any NPZ testing data (data not shown).

### IRF-8 Density in Monocytes Correlates With Brain Volumes

**Figure 3:**
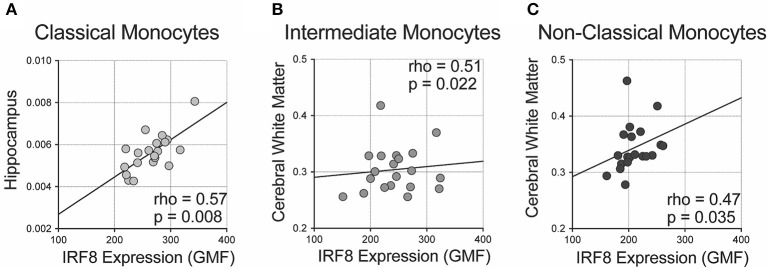
Correlations between IRF-8 expression on myeloid cells and regional brain volumes. Correlations between IRF-8 expression of **(A)** classical, **(B)** intermediate, and **(C)** non-classical monocytes and regional brain volumes corrected for intracranial volume.

We analyzed correlations of IRF-8 density with regional brain volumes (Figure 3 and Supplementary Table 3). Higher IRF-8 expression in classical monocytes correlated with larger ICV-adjusted hippocampal volume (rho = 0.573; *p* = 0.008; Figure 3A). Higher IRF-8 expression in intermediate and non-classical monocytes correlated with larger cerebral white matter volume corrected for ICV (rho = 0.509; *p* = 0.022 and rho = 0.473; *p* = 0.035, respectively; Figures 3B,C). DC IRF-8 expression did not correlate with regional brain volumes (Supplementary Table 3). Age did not correlate with volumes of hippocampus or cerebral white matter (data not shown).

### IRF-8 Density in Myeloid Cells Correlates With Plasma Inflammatory Mediators

**Figure 4:**
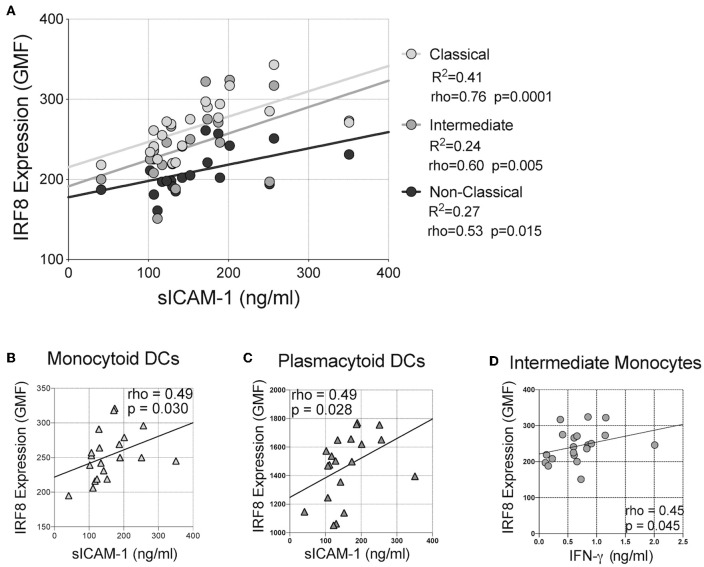
Correlations between IRF-8 expression on myeloid cells and soluble inflammatory markers. Correlations between IRF-8 expression of **(A)** classical, intermediate, and non-classical monocytes, **(B)** myeloid and **(C)** plasmacytoid DCs and soluble intercellular cell adhesion molecule-1 (sICAM-1). **(D)** Correlation between IRF-8 expression in intermediate monocytes and plasma interferon-gamma (IFN-γ) level.

Finally, correlations of IRF-8 density and plasma cytokine levels were analyzed (Figure 4 and Supplementary Table 4). Higher IRF-8 density in classical, intermediate and non-classical monocytes all correlated with higher sICAM-1 levels (rho = 0.756; *p* = 0.0001; rho = 0.600, *p* = 0.005 and rho = 0.534, *p* = 0.015, respectively; Figure 4A). Higher IRF-8 density in mDCs and pDCs also correlated with higher sICAM-1 (rho = 0.490, *p* = 0.030; rho = 0.490, *p* = 0.028, respectively; Figures 4B,C). Higher IRF-8 density in intermediate monocytes also correlated with higher IFN-γ levels (rho = 0.453; *p* = 0.045; Figure 4D). No correlations between IRF-8 and the other measured inflammatory mediators were observed (Supplementary Table 4). However, separately, higher sICAM-1 levels correlated with higher learning memory NPZ scores (rho = 0.484, *p* = 0.036) (Supplementary Table 5). Higher IL-8 and MMP-9 levels correlated with lower NPZ_LM scores (rho = −0.523, *p* = 0.022) and NPZ_WM (rho = −0.502, *p* = 0.024) scores, respectively (Supplementary Table 5). Actual values of NPZ scores and soluble inflammatory markers are shown in Supplementary Tables 6, 7, respectively.

## Discussion

Given our intriguing methylation data demonstrating a link between the *IRF8* gene and HIV-associated cognitive impairment, the protein expression of intracellular IRF-8 was investigated in peripheral myeloid cells in virally suppressed HIV-infected individuals with multi-dimensional measures of cognitive performance and brain volumes. Although constitutive IRF-8 expression has been previously reported in myeloid cells, to our knowledge, our data are the first to link reduced IRF-8 protein expression in monocytes and DCs to worse NP testing performance and smaller brain volumes in virally suppressed HIV-infected individuals, suggesting this transcription factor is relevant in the neuropathology of brain health in treated HIV possibly by secretion of cytokines beneficial for CNS function and control of proviral quiescence of HIV.

Both in humans and mice, *IRF8* gene mutations are associated with depletions in circulating monocytes and dendritic cells (42) with IRF-8 driving myeloid differentiation. The maintenance of the monocytic homeostatic pool is not affected by deletion of *IRF-8* in mice (23, 43, 44). In fact, in our cohort, total and subset myeloid, both monocyte and DC, frequencies were not affected by IRF-8 expression (data not shown).

The functional consequences of higher IRF-8 expression as well as the relationship to CNS measures merits further study. Indicative of systemic inflammation, the presence of inflammatory cytokines can drive IRF-8 expression, which may further influence functional differences in monocyte and DC subsets (45–47). Indeed, IRF-8 is essential for controlling infection in both mice and humans (48, 49). Mutations in *IRF8* are associated with increased susceptibility to *M. tuberculosis* infection (47). Moreover, IRF-8 is crucial for the optimal activation of NLRC4 inflammasome and deletion of the gene induce susceptibility to *Salmonella Typhimurium* and *Burkholderia thailandensis* in mice (49). Finally, in the context of viral control, IRF-8 in the cytosol is crucial for NF-kB activation via the TLR9-MyD88 dependent pathway (50). IRF-8 also acts as a transcription factor for the induction of *Il12b* gene upon TLR stimulation (51, 52). In addition, monocyte cytokine responses to pathogenic stimuli, such as interferon-beta (IFN-β) secretion, can also be synergistically regulated by IRF-8 and IRF-3 binding of the ETS/IRF composite element of the IFN-β promoter (53).

Although inflammatory responses are historically viewed as undesirable in the context of pathological conditions, recent studies, on the other hand, have shown the importance of inflammatory responses that bridge subsequent anti-inflammatory responses, which suggests complicated nature of inflammation (54, 55). In HIV-infected individuals, a previous report highlighted positive correlations of higher IFN-γ, MCP-1, and TNF-α level to higher volumes of the putamen, pallidum and amygdala, whereas higher IL-1β, IL-6, and IL-18 plasma levels were related to lower volumes of the putamen, pallidum, thalamus, hippocampus, and amygdala (56). In line with this report, our data showed the positive correlation between IRF-8, which expression is driven by cytokines including IFN-γ, and brain volume. In a cutaneous wound healing murine model, the down-regulation of IRF-8 by *in vivo* miRNA-induced silencing complex administration decreased transcription of inflammatory mediators associated with M1 macrophages (IL-1β, IL-6, iNOS, TNF-α) and impaired wound healing at the site of injury (57). Yuan et al. has previously reported that plasma soluble ICAM-5 level was increased in HIV-infected subjects with cognitive impairment compared to HIV-infected subjects with normal cognition (58). Since sICAM-5 suppresses immune activation status (58) whereas sICAM-1 activates the immune system, this supports our results (59). Intriguingly, we found a correlation such that higher plasma sICAM-1 levels correlate with better learning memory NPZ scores (Supplementary Table 5). Furthermore, our findings point toward an interesting correlation found among MMP-9 and learning memory NPZ score such that higher MMP-9 correlated with lower learning memory NPZ scores (Supplementary Table 5). Similarly, higher IL-8 negatively correlated with working memory scores (Supplementary Table 5). MMP-9 has previously been described as inhibiting IL-23 mediated pro-inflammatory responses in dendritic cells while IL-8 is essential for mounting a sufficient T helper cell response (60–62). It has been described that IRF-8 is involved in CCL4 expression which in turn promotes MMP-9 expression in macrophages (63). Additional studies into networked IRF-8, MMP-9, IL-23, and IL-8 activities to decipher the nature of inflammation in people living with HIV are warranted.

Additionally, IRF-8 activity may regulate HIV provirus production. *In vitro* HIV infection of Jurkat cells has been shown to significantly increase IRF-1 gene expression (42). However, when HIV-infected Jurkat cells were stably transfected with IRF-8, p24 production was decreased compared to the control vector, due to the ability of IRF-8 to inhibit the binding of IRF-1 to Tat, suggesting that IRF-8 is a dominant negative regulator of IRF-1 activity and can block HIV-1 transcription (42). When the chronically HIV-infected pro-monocytic cell line, U1, was treated with a histone deacetylase inhibitor, a concomitant decrease in IRF-8 gene and protein expression was observed along with an increase in gag gene expression (64). This decreased expression of IRF-8 following reactivation of latency suggests a role of IRF-8 in maintaining of proviral quiescence of HIV. Higher IRF-8 levels in the myeloid cells may permit better control of HIV levels within the monocytes which may be a principle mechanism that may maintain viral quiescence and limit neuroinflammation leading to CNS injury manifesting as cognitive impairment (65).

Our observed associations between IRF-8 density and both cognitive performance and regional brain volumes are supported by functional relationships between the relevant regions and NP domains. Consistent with its relationship to better working memory and learning and memory, higher IRF-8 expression in classical monocytes correlated with larger volumes of hippocampus. The involvement of the hippocampus in working memory is well-known (66): in Alzheimer's disease, decline in this domain is associated with hippocampal atrophy (67), as is learning and memory impairment (68). Hippocampal volume has also been positively correlated with memory performance in healthy young individuals (69). The active role played by white matter in learning and memory (70) supports the correlations of IRF-8 expression in intermediate and non-classical monocytes with cerebral white matter volume and with learning and memory. Similarly, correlations of non-classical monocyte expression of IRF-8 with cerebral white matter volume, working memory, executive function, and global NP performance are in line with the literature relating white matter reductions to cognitive decline (71).

Correlations linking the periphery and brain might reflect monocytes migrating into the tissues and carrying out a protective role with regards to HIV infection. IRF-8 expression in monocytes and DCs tracking with NP performance suggests a role for IRF-8 in HIV-related cognitive dysfunction and may highlight a potential therapeutic target. In Vogt-Koyanagi-Harada disease (VKH), monocyte-derived dendritic cells (MDDCs) from active VKH patients have decreased IRF-8 mRNA expression in association with higher methylation levels compared with normal controls or individuals with inactive VKH. Treating DCs the demethylation reagent, 5-Aza-2'-deoxycytidine (DAC), increased IRF-8 mRNA expression by reducing the methylation level of the IRF-8 gene (72). Hypermethylation of IRF-8 in myeloid cells in the context of VKH is similar to our previously published methylation data in HIV (21). Pharmacological interventions targeting demethylation of IRF8 may offer a novel strategy in the context HIV-associated cognitive dysfunction. This may be feasible with the use of microRNA interference as evidenced by murine studies that have enriched pDC frequencies with administration of miR-103 (73). An evaluation of IRF-8 regulatory activity and characterization of affected genomic elements will be beneficial to understand precise mechanisms of IRF-8 function in myeloid cells. Lin et al. demonstrated identification of DC lineage-specific transcription factor regulatory networks for IRF-8 with studies examining consecutive changes of stage-specific expression for key DC regulators are associated with specific histone modifications in promoter and enhancer sequences (74).

Here we report the lowest levels of IRF-8 in peripheral myeloid cells from virally suppressed individuals with chronic HIV correlates with worse cognitive performance. The conclusions of this study are limited by the small sample size of only 20 participants. In addition, the design of this study did not include an HIV-uninfected control group, which limits our ability to conclusively define a causal link between IRF-8 expression and HIV infection. Future longitudinal studies are also necessary to examine the causal relationships between the high IRF-8 expression and HIV-related cognition dysfunction.

## Data Availability Statement

All datasets generated for this study are included in the article/Supplementary Material.

## Ethics Statement

The study, which has been previously described (25), was approved by the University of Hawaii Manoa Committee on Human Studies. The patients/participants provided their written informed consent to participate in this study.

## Author Contributions

MD'A, KK, MC, DO-A, DC, CS, and LN conceived and designed the study. MD'A, KK, and TP performed the experiments. MD'A, KK, TP, TF, EL, and VK analyzed the data. MD'A, TF, and EL wrote the manuscript.

### Conflict of Interest

The authors declare that the research was conducted in the absence of any commercial or financial relationship that could be construed as a potential conflict of interest.

## Supplementary Material

Supplementary Figure 1 Representative gating strategy for T cells, monocytes, dendritic cells using flow cytometry. After excluding doublets and dead cells, CD4^+^ and CD8^+^ T cells were identified from the CD3^+^ cell population. From the CD3^−^ population, after the exclusion natural killer (NK) and B cells (CD7, CD19, or CD20 positive cells), monocytes, positive for HLA-DR and CD11b, were subset by CD14 and CD16 expression: Classical monocytes (CD14^++^CD16^−^), intermediate monocytes (CD14^++^CD16^+^) and non-classical monocytes (CD14^+^CD16^+^). Dendritic cells (DCs; CD3^−^ CD7^−^ CD19^−^ CD20^−^ CD14^−^ HLA-DR^+^) were subset into myeloid (CD11c^+^) or plasmacytoid (CD123^+^) DCs. IRF-8 expression was then assessed on T cells subsets (CD4^+^ and CD8^+^), DC subsets and monocyte subsets.

Supplementary Figure 2 IRF-8 density in each myeloid subset from HCV+ and HCV- subjects. Intracellular IRF-8 expression in **(A)** monocyte each subset and **(B)** DC each subset from HCV+ and HCV- subjects.

Supplementary Table 1 Frequencies of each myeloid subset. After performing gating strategy as detailed in Supplementary Figure 1, frequencies of myeloid subsets with respect to total PBMCs are given here.

Supplementary Table 2 Complete analyses of correlations of IRF-8 density and neuropsychological testing correlation. Correlations using Kruskal Wallis testing was used to evaluate associations between IRF-8 expression density and measures of neuropsychological tests, including: learning memory, working memory, executive function, psychomotor, and a composite neuropsychological score of the four domains referred to as global. The values reflected in Figure 2 are reflected here in table format as this table.

Supplementary Table 3 Complete analyses of correlations of IRF-8 density and regional brain volumes. Correlations using Kruskal Wallis testing was used to evaluate associations between IRF-8 expression density and regional brain volumes listed in the table. Values reflected in Figure 3 are reflected here in table format as this table.

Supplementary Table 4 Complete analyses of correlations of IRF-8 density and soluble inflammatory markers. Correlations using Kruskal Wallis testing was used to evaluate associations between IRF-8 expression density and soluble inflammatory markers, including: soluble E-Selectin (sE-Selectin), soluble vascular cell adhesion protein 1 (sVCAM-1), soluble intercellular adhesion molecule 1 (sICAM-1), metallomatrixprotein-9 (MMP-9), myeloperoxidase (MPO), plasminogen activator inhibitor-1 (tPAI-1), C-reactive protein (CRP), serum amyloid A (SAA), serum amyloid P component (SAP), interleukins (IL-1β, IL-6, IL-8, IL-10), Tumor necrosis factor alpha (TNF-α), monocyte chemoattractant protein-1 (MCP-1), vascular endothelial growth factor (VEGF), interferon gamma (IFN-γ). Values reflected in Figure 4 are reflected here in table format as this table.

Supplementary Table 5 Complete analyses of correlations of neuropsychological testing and soluble inflammatory markers. Correlations using Kruskal Wallis testing was used to evaluate associations between neuropsychological testing scores and regional soluble inflammatory markers listed in the table.

Supplementary Table 6 Values of NPZ scores in each subject. Actual values of NPZ scores in each subject are shown in the table.

Supplementary Table 7 Values of soluble inflammatory cytokine levels in each subject. Actual values of soluble inflammatory cytokine levels measured in each subject are shown in the table.
