# Supplementary figures and images for: Lower Interferon Regulatory Factor-8 Expression in Peripheral Myeloid Cells Tracks With Adverse Central Nervous System Outcomes in Treated HIV Infection
Source: Front Immunol. 2019 Nov 29;10:2789. doi: 10.3389/fimmu.2019.02789 (PMC6895026; doi:10.3389/fimmu.2019.02789)

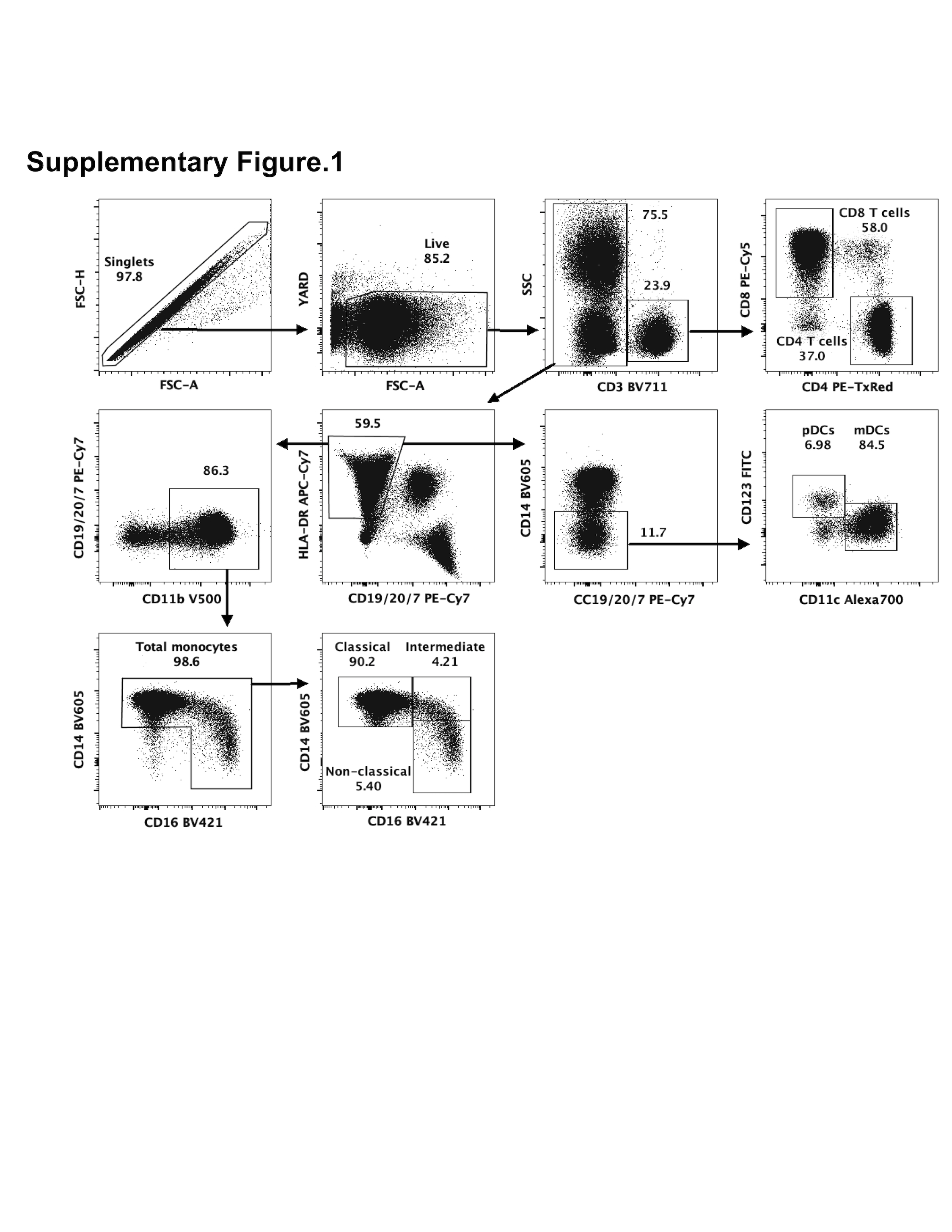

Supplement: Supplementary Figure 1 — Representative gating strategy for T cells, monocytes, dendritic cells using flow cytometry. After excluding doublets and dead cells, CD4+ and CD8+ T cells were identified from the CD3+ cell population. From the CD3− population, after the exclusion natural killer (NK) and B cells (CD7, CD19, or CD20 positive cells), monocytes, positive for HLA-DR and CD11b, were subset by CD14 and CD16 expression: Classical monocytes (CD14++CD16−), intermediate monocytes (CD14++CD16+) and non-classical monocytes (CD14+CD16+). Dendritic cells (DCs; CD3− CD7− CD19− CD20− CD14− HLA-DR+) were subset into myeloid (CD11c+) or plasmacytoid (CD123+) DCs. IRF-8 expression was then assessed on T cells subsets (CD4+ and CD8+), DC subsets and monocyte subsets. [file Image_1.TIFF]

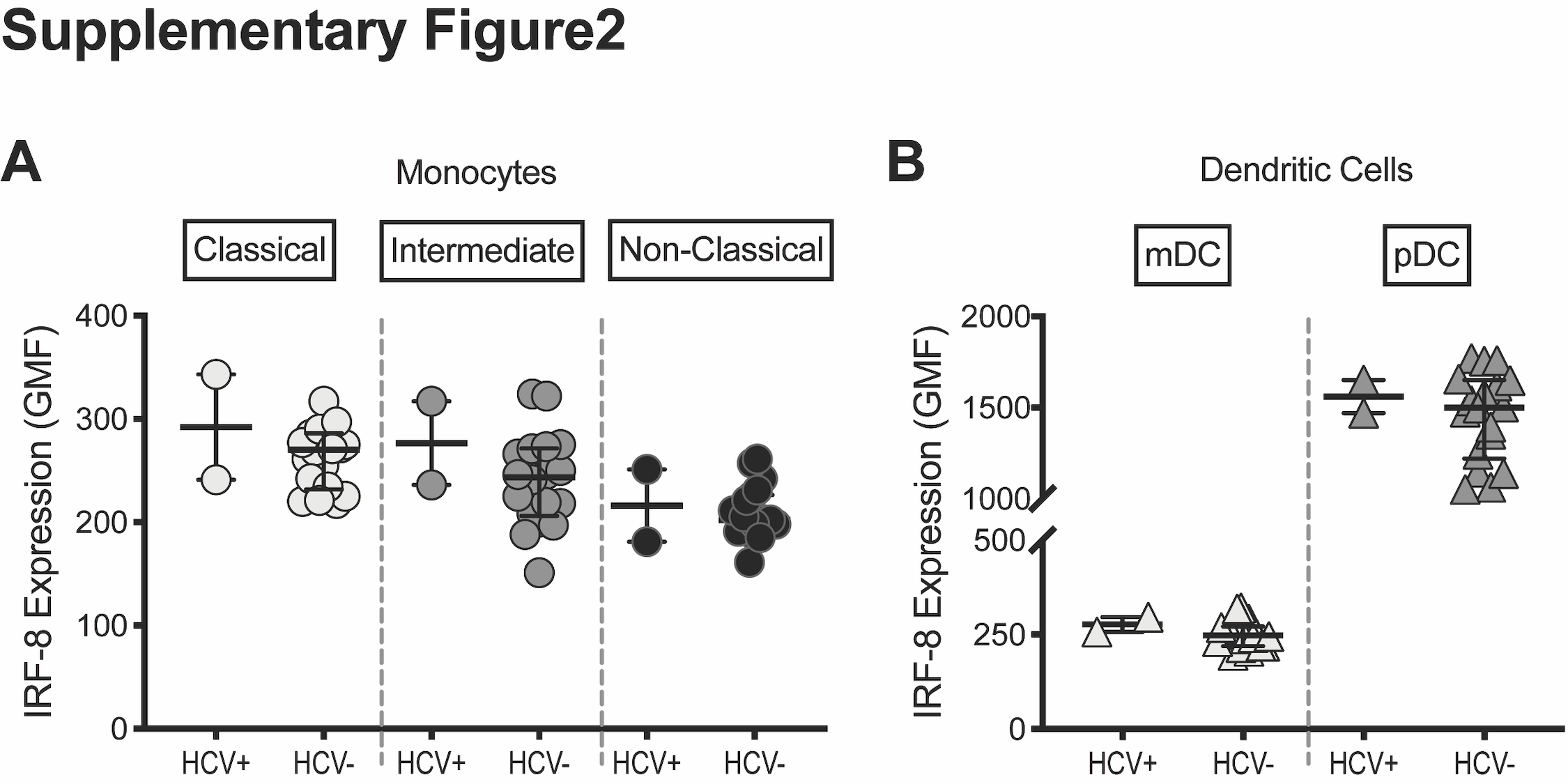

Supplement: Supplementary Figure 2 — IRF-8 density in each myeloid subset from HCV+ and HCV- subjects. Intracellular IRF-8 expression in (A) monocyte each subset and (B) DC each subset from HCV+ and HCV- subjects. [file Image_2.TIF]
